# Prevalence of Gastrointestinal Parasitic Infections and Assessment of Deworming Program among Cattle and Buffaloes in Gampaha District, Sri Lanka

**DOI:** 10.1155/2018/3048373

**Published:** 2018-10-09

**Authors:** Nayana Gunathilaka, Dimuthu Niroshana, Deepika Amarasinghe, Lahiru Udayanga

**Affiliations:** ^1^Department of Parasitology, Faculty of Medicine, University of Kelaniya, Ragama, Sri Lanka; ^2^Department of Zoology and Environment Management, Faculty of Science, University of Kelaniya, Dalugama, Sri Lanka; ^3^Department of Biosystems Engineering, Faculty of Agriculture and Plantation Management, Wayamba University, Makandura, Sri Lanka

## Abstract

Gastrointestinal (GI) parasitic infection is a serious issue in cattle management. The effects of GI parasites may vary with age, sex of cattle, nutritional condition, and severity of infection. Prevalence of GI parasites among cattle population in Gampaha District has not been studied and there is no published study available. A total of 45 farms rearing cattle were selected randomly in three areas, namely, Kelaniya, Ganemulla, and Welisara, under three Veterinary Surgeon Divisions (VSD) in Gampaha District (Mahara, Gampaha, and Welisara). Freshly voided cattle fecal samples were collected randomly from the selected farms during March 2017–December 2017. Out of 163 cattle and buffaloes examined, 13.39% (n=22) were positive for eggs of one or more species of GI parasites. The prevalence of parasitic infection was higher in buffaloes (31.25%, 5/16) as compared to that of cows (11.56%, 21/147), but the difference was not significant (*P* >0.05). Hookworms (*Bunostomum* spp.), whipworms (*Trichuris* spp.), digenetic trematodes (*Paramphistomum* spp.), cestodes (*Moniezia *spp.), and oocysts of protozoans (coccidians) were found during the study. The nontreated animals indicated the highest percentage of parasitic infections accounting for 46.67% (n= 14), followed by partially treated individuals (15.15%, n= 5). GI parasite prevalence in males was higher when compared to that of females, but the difference was nonsignificant (*P* >0.05). General Linear Modelling (GLM) revealed that the effect of treatment status was significantly associated with the prevalence of GI parasites. The calves and yearlings had the highest rate of GI parasitic infections. The highest infection rate was observed at Kelaniya, followed by Welisara. Future investigations are necessary to evaluate the economic impact of GI parasites in the study areas.

## 1. Introduction

Livestock farming, particularly rearing of cattle (*Bos indicus/Bos tarsus*) and Ceylon buffalo (*Bubalus bubalis migona*), is traditionally practiced by rural people in Sri Lanka [1]. Rearing of cattle in the country is catering for draught power, milk production, and meat production. Buffaloes are predominantly used for farm power in the cultivation of rice as well as production of curd [1].

Parasitic diseases caused by intestinal parasites constitute a major impediment to livestock production [2]. All ages of cattle are affected by a diverse set of intestinal parasites. These infections are rarely associated with high mortality of cattle. However, their effects are usually characterized by lower outputs of animal products, byproducts, manure, and traction, thereby affecting the contributions of cattle in ensuring food security, especially in developing countries [2, 3]. The productivity losses through reduced feed intake and decreased efficiency in feed utilization due to subclinical or chronic infections are responsible for economic losses in the livestock industry [4].

In addition, these infections enhance susceptibility to bacterial and viral diseases and losses resulting from condemnation of carcasses and organs, as well as cost of drugs and veterinary care [5]. Gastrointestinal parasites like coccidian, ascarid, strongyle,* Setaria*, and amphistomes were documented in countries with tropical and temperate climatic conditions such as India, Bangladesh, South Africa, Sri Lanka, Italy, and Mongolia, with a prevalence rate ranging from 20 to 96% [6–11]. Some studies conducted in Sri Lanka have recorded concurrent helminthic and coccidial infections at a rate of 78% among the cattle [7].

Anthelmintics and antiprotozoal agents have been used to control gastrointestinal parasitic infections over the last ten decades [12]. They have succeeded in reducing intestinal parasitic infections, but none has been able to diminish the reinfestation of diseases [13]. However, excessive use of anthelmintic drugs has led to developing of anthelmintic-resistant parasites, which are being reported from many parts of the world. Further, it has resulted in a fear of anthelmintic residues in the milk and meat of livestock animals [14].

In order for an anthelmintic strategy to be successful, in-depth knowledge of pathophysiology and epidemiology of the parasite, in the context of immunity and management of the host, is required. Therefore, periodical monitoring of parasitic species among livestock animals would be beneficial to control and manage diseases at early stages of infections in farm management practices.

Prevalence of gastrointestinal parasites among cattle population in Gampaha District has not been studied and there is no published study available. In addition, it is important to study the present situation of parasitic infections in cattle and associated risk factors. Hence, the aim of the present study was to determine the prevalence of single and concurrent infections of GI parasites among cattle and the intensity of infections in selected farms in Gampaha District of Sri Lanka.

## 2. Material and Methods

### 2.1. Study Area

The District of Gampaha, located in the Western Province of Sri Lanka covering an area of 1,387 km^2^, was selected as the major study area. The mean annual rainfall of Gampaha remains around 2,398 mm, while the mean annual temperature is about 27.3°C.

### 2.2. Selection of Sampling Locations

**Figure 1:**
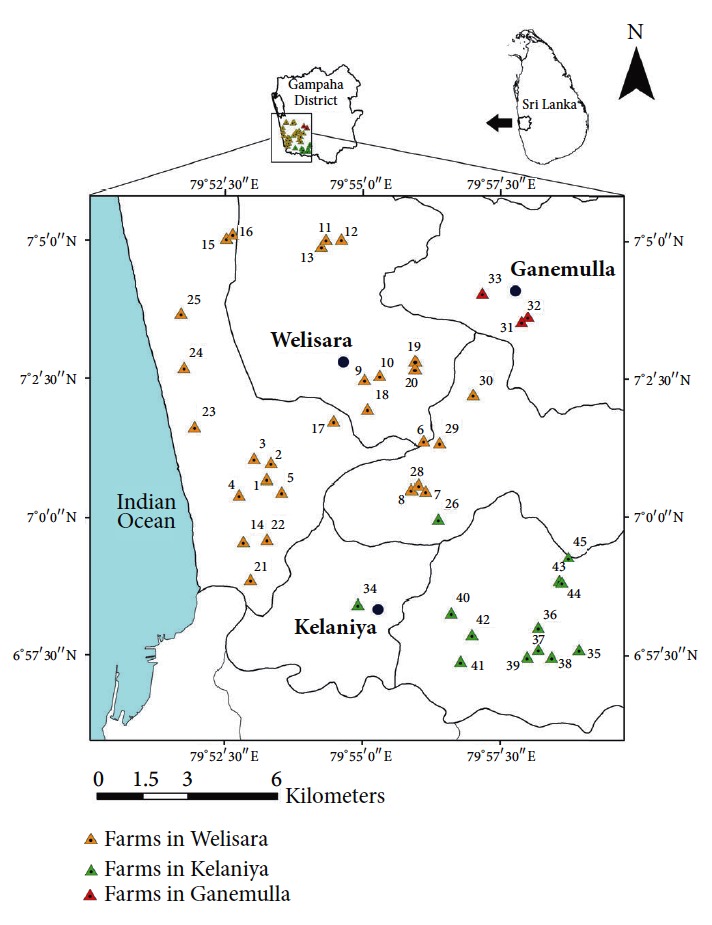
Sampling sites located within the three Veterinary Surgeon Divisions in Gampaha District.

Based on the registered cattle farms at Veterinary Investigation Centre, Welisara, 45 farms were selected randomly for the study using a random number table method based on the geographic location. These 45 farms fell into three Veterinary Surgeon Divisions (VSD), namely, Mahara VSD, Gampaha VSD, and Welisara VSD. Mainly these farms were distributed in Kelaniya, Ganemulla, and Welisara areas. The geographical distribution of the selected farms is illustrated in Figure 1. The farms that refused to participate in the present study were excluded and replaced with others in the same area.

### 2.3. Treatment Procedure and Categorization of Treatment Status

The treatment against gastrointestinal (GI) parasites in calves was administered at 21-day, 3-month, 6-month, and 12-month intervals, respectively, with a standard mixture of Albendazole and Fenbendazole, in accordance with the country guidelines based on the body mass of cattle. Subsequently, a high dosage is provided annually as the deworming practice. Cattle that underwent the above standard procedure were considered as “treated,” while cattle that missed two or more treatments were grouped as “partially treated.” The rest of the population that were never treated were considered as the “nontreated” sample in the present study.

### 2.4. Collection and Processing of Fecal Samples

A total of 163 freshly voided cattle fecal samples (30 g) from the selected farms were collected randomly into 275 ml sterile plastic containers with a screwed lid directly from the rectum of the cattle or freshly dropped feces from the ground separately, over a period of nine months from March 2017 to December 2017.

Each container was labeled assigning a reference number. About 15 ml of 10% formalin was introduced* in situ* to each collected stool sample in order to prevent embryonation of the parasitic eggs. The preserved samples were transported to the laboratory at the Department of Zoology and Environment Management, Faculty of Science, University of Kelaniya, Sri Lanka, under cold conditions in Rigifoam boxes with ice cubes. The samples were stored in a bottle cooler at 4°C, until used for parasitological examination. Information, such as age and sex of the cattle, status of management, and deworming practices of the farm, was collected by interviewing the farmer and from the area's veterinary surgeon.

### 2.5. Sample Preparation for Parasitological Screening

Fecal samples were analyzed using standard parasitological screening techniques for intestinal parasites, namely, simple salt floatation technique followed by sedimentation [15], direct saline and iodine smear observations.

### 2.6. Morphological Identification and Quantification

The parasite eggs/oocysts, larvae, and cysts were examined and identified to the generic level of the parasite by microscopy based on the morphological identification keys described by Zajac and Conboy [16]. Further, length and width of each identified parasitic stage were measured using OPTIKA Microscope. For quantitative analysis, the modified McMaster technique was used to estimate eggs/oocysts per gram of feces (epg/OPG) as described in the following equation by RVC/FAO Guide to Veterinary Diagnostic Parasitology.

(1) Eggs/oocysts  per  1gram  of  feces=∑Ni+∑Nii x 50,

where** Ni** is number of parasitic stages in chamber 1 and **N** ^**ii**^ is number of parasitic stages in chamber 2.

### 2.7. Statistical Analysis

All the data were entered into a Microsoft Excel worksheet and all the data analysis was performed using IBM SPSS Statistics (version 23 copyright IBM Corporation). The significance of the effect of the treatment status, spatial location, age, gender, and type of cattle on the prevalence of GI parasitic infections among cattle was statistically evaluated by using the General Linear Model (GLM) followed by Tukey's pairwise comparison in SPSS (version 23). In addition, the Bray-Curtis similarity based cluster analysis followed by analysis of similarities (ANOSIM) (i.e., a nonparametric analog of MANOVA) was utilized to identify the overall clustering status of cattle from different areas in terms of the prevalence of different parasite families [17].

Further, the Principal Coordinates (PCO) analysis and Distance-Based Redundancy Analysis (dbRDA) were also performed to highlight and visually represent the underlying segregation patterns of the study populations based on variations in the GI parasite abundance using the Plymouth Routines in Multivariate Ecological Research version 6 (PRIMER 6).

## 3. Results

### 3.1. Treatment Status of Cattle against Intestinal Parasites

The study population was comprised of 100 “treated,” 33 “partially treated,” and 30 “nontreated” individuals. The parasitological observations confirmed that the immunity against gastrointestinal (GI) parasitic infections increased along with the effectiveness of the treatment status. The nontreated animals indicated the highest percentage of parasitic infections accounting for 46.67% (n= 14), followed by partially treated individuals (15.15%, n= 5).

Meanwhile, the treated population was denoted by only 3% (n= 3) occurrence of GI parasitic infections (Table 1). As indicated in General Linear Modelling (GLM), the effect of treatment status was significantly correlated with the prevalence of GI parasitic infections (*P*<0.05 at 5% level of significance) in the study population. The results of post hoc analysis (Tukey's pairwise comparison) confirmed that the GI infection rates differed significantly among cattle in three treatment categories (Table 1).

### 3.2. Prevalence of Parasites in Cattle Population

**Figure 2:**
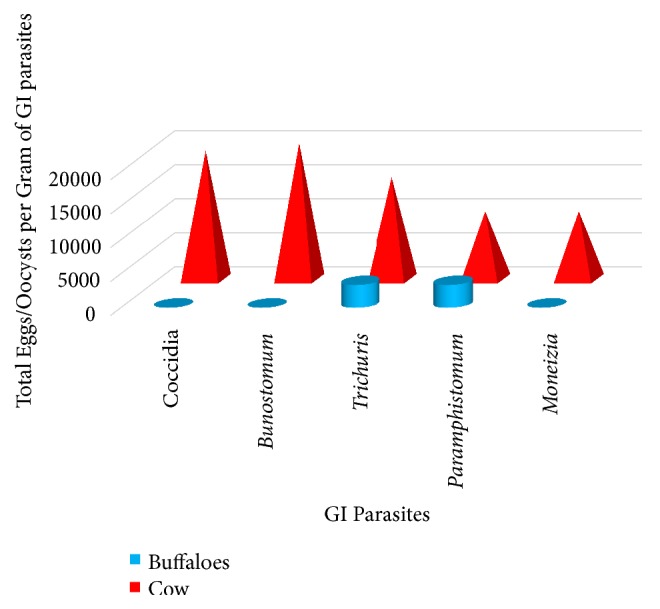
Variation of the total eggs per gram of GI parasites among the cows and buffaloes.

A variety of GI parasites, namely, eggs of hookworms (*Bunostomum* spp.), whipworms (*Trichuris* spp.), amphistomes, cestodes (*Moniezia* spp.), and oocysts of protozoans (coccidians), were found within the study population of cattle. The parasite-wise prevalence of GI parasites in cows and buffaloes is illustrated in Table 2. In general, the total number of eggs or oocysts found in cows was relatively higher than that of buffaloes (Figure 2).

**Figure 3:**
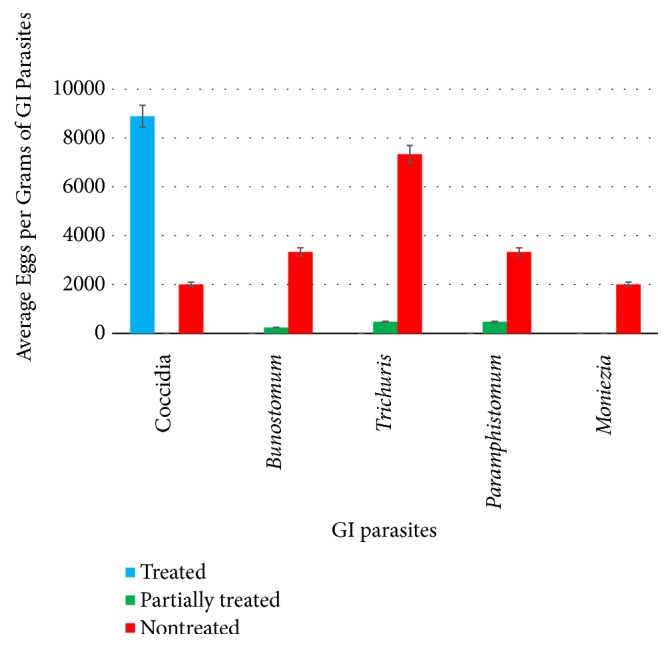
Variation of the total eggs per gram of GI parasites among the cattle with different treatment status.

Only coccidian oocysts were found among the treated sample of cattle indicating that these parasites may have a higher tolerance range against standard treatment protocols (Figure 3). In case of the partially treated sample, eggs of both hookworms and* Trichuris* spp. were found. Nontreated cattle were infected with all the five types of GI parasites including the above species along with amphistomes and* Moniezia* spp. (Figure 3).

Based on the above, the present study indicated very high parasitic infections in cattle for nematode and coccidial oocysts. The average FEC of* Trichuris* spp.,* Bunostomum* spp., and coccidian oocysts were noted as 7333 epg, 3333 epg, and 2000 epg, respectively, especially in the farms which were not having a proper treatment and investigations. However, cattle reared under the investigation of the Veterinary Investigation Centre also showed an average count of 8888 coccidial oocysts per gram of feces, but they were negative for nematode parasites (Figure 3).

### 3.3. Type and Gender Precise Prevalence of Parasites

A total of 16 buffaloes and 147 cows were examined during the current study. Of them, 31.25% (n=5) of buffaloes were infected with GI parasites (Table 3), indicating a higher susceptibility rate than cows, which had an infection rate of 11.56% (n=17). However, as emphasized above, the total egg/oocyst amount found in infected cows was relatively higher than buffaloes.

In case of gender, 32.14% (n=9) of the male cows were infected, while only 6.72% (n=8) of females indicated the presence of any GI parasite. A similar trend was observed in buffaloes also, whereby 60.0% (n=3) of males were infected. In general, out of 33 males, 12 were infected with GI parasites, denoting that males had a higher prevalence rate of GI parasites (Table 3).

The GLM advocated that males had a significantly higher susceptibility to GI parasites than females (*P*<0.05), while buffaloes had a significantly higher prevalence of GI parasites than cows (*P*<0.05, at 5% level of significance). Interestingly, the interaction among types and genders on the prevalence of parasites was also significant (*P* =0.04), in accordance with the results of GLM.

Amphistome eggs present in male buffaloes (18.75%) were higher than the female buffaloes.* Trichuris *spp. eggs were found in equal amounts among both male and female buffaloes. Oocysts of Coccidia (2.04%) and eggs of* Trichuris *spp. (3.4%) were more in male cows than the female cows. Eggs of hookworms and amphistomes were observed in both male and female cows equally, while eggs of* Moniezia *spp. were found only in female cows (0.68%).

It was interesting to note that even though all the abovementioned parasites were found in the infected population of cows, only* Paramphistomum* and* Trichuris* spp. were found in buffaloes (Figure 2). The results of the GLM confirmed that the prevalence rates of GI parasites differed among the cows and buffaloes (*P *< 0.05).

### 3.4. Age-Wise Prevalence of Gastrointestinal Parasites

The cattle population was defined as calve (≤ 12 months), yearling/heifer (13–60 months), and elderly/matured (>60 months) based on their life span. In cows, the calves (17.07%) had a higher susceptibility towards GI infections followed by yearling (14.29%). Interestingly, the elderly cows had the lowest infection rate of 4.0% (Table 4). Among buffaloes, yearlings (50%) had the highest rate of infections, followed by calves (33.33%). The effect of age on the prevalence of GI parasites was also significant as indicated by the GLM (*P* <0.05 at 95% level of confidence). Surprisingly, the combined effect of age and type was not statistically significant on the susceptibility of the cattle (*P* >0.05) in accordance with the test statistics of GLM (Table 4).

### 3.5. Effect of Spatial Location on the Parasitic Infection

The study population included 163 cattle selected from three study areas, namely, Welisara (n=118), Kelaniya (n=35), and Ganemulla (n=10), covering 45 farms. The highest infection rate of GI parasites was observed at Kelaniya (31.43%), followed by Welisara (9.32%). Interestingly, none of the cattle from the Ganemulla area were infected with any GI parasite. In case of the diversity of the GI parasites, all the observed parasites except for amphistomes were found from the cattle in the Welisara area. Subsequently,* Moniezia *spp. were not found from the cattle population in the Kelaniya area, which were screened for intestinal parasites (Table 5).

**Figure 4:**
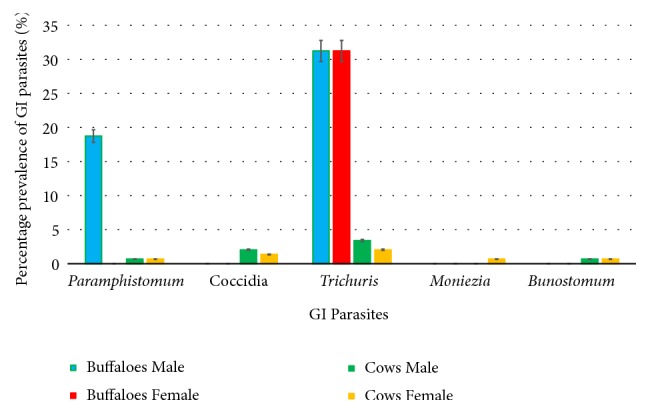
Dendrogram showing the spatial clustering of studied sites based on the prevalence of GI parasitic infections among cattle.

As indicated by the results of GLM, the spatial location of the cattle significantly affected the incidence of GI parasites in cattle (*P* =0.02, at 95% level confidence). The overall clustering status of cattle from different areas in terms of the prevalence of parasite types is illustrated in Figure 4.

**Figure 5:**
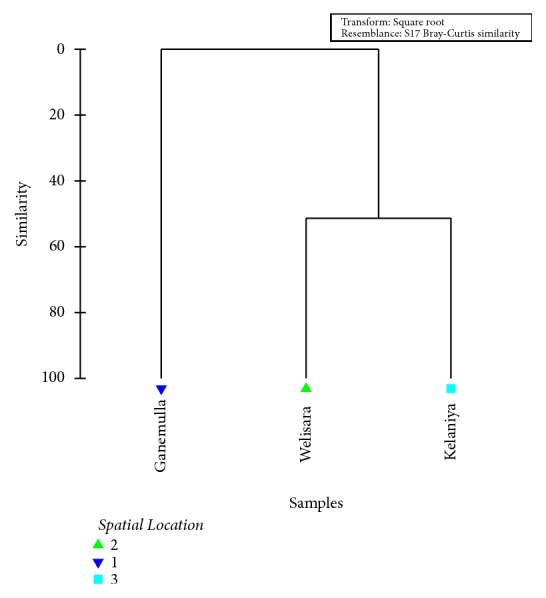
Ordination of the study sites based on PCO_1_ and PCO_2_ scores of PCO analysis based on the prevalence of GI parasitic infections among cattle.

As indicated by the Bray-Curtis similarity clustering, both Kelaniya and Welisara share a similarity of 51.4 % in terms of the prevalence and diversity of GI parasites among cattle. Meanwhile, cattle from Ganemulla remain isolated from the above cluster (Figure 5). The global R value of 0.92 gained for the analysis of similarities (ANOSIM) also confirmed the above observation at a significance level of 5%.

Meanwhile, both PC_1_ (84.1 %) and PC_2_ (15.9 %) axes of the Principal Coordinates (PCO) that accounted for the total variation (100%) of the GI parasite prevalence among the studied cattle population suggested the emergence of two major clusters as Kelaniya and Welisara together, while Ganemulla remained isolated, confirming the above observations (Figure 5).

**Figure 6:**
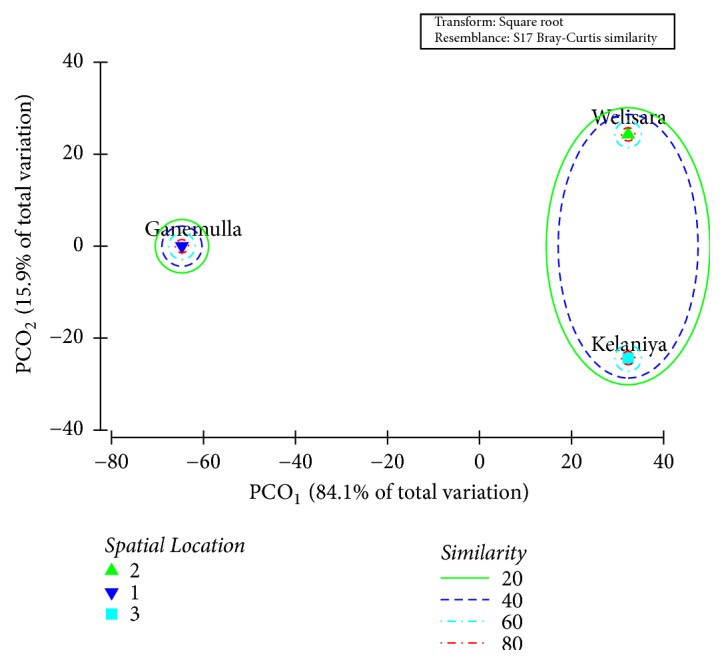
dbRDA plot depicting spatial variation of cattle belonging to different study sites based on the prevalence of GI parasites.

Further the radiating axes from the center of the Distance-Based Redundancy Analysis (dbRDA) plot clearly denoted that the cattle in Welisara area are dominated by the prevalence of Coccidia,* Trichuris* spp., and* Moniezia* spp. as GI parasites, while hookworms and* Paramphistomum* spp. are prominent among the cattle in Kelaniya (Figure 6). The results of the post hoc analysis (Tukey's pairwise comparison) also confirmed the above deduction by indicating that the GI infection rates differ significantly among cattle belonging to the three study areas (*P* <0.05).

### 3.6. Factors Affecting the Gastrointestinal Parasitic Infections

As denoted by the General Linear Modelling (GLM), the treatment status, spatial location, age, gender, and type of cattle were recognized as significant parameters that affected the incidence of GI parasites among cattle. Further, the combined effects of treatment status, spatial location, age, and gender were also significantly influencing the GI parasitic infection rates in cattle as interactive effects (*P* <0.05 at 95% level of confidence).

## 4. Discussion

Control of gastrointestinal parasitic infections in animals requires a comprehensive knowledge of the disease epidemiology and understanding of the pasture management, farm management practices, and agroclimatic conditions such as temperature and rainfall [6]. The numbers of parasitic eggs and coccidial oocysts developed inside the host animals vary depending on the parasite species, level of host susceptibility, the health status of the animal, and immunological status [6, 18].

During this study, parasitic stages of five different parasites were detected in the fecal samples. The identified parasitic stages were eggs of hookworms (*Bunostomum* spp.), whipworms (*Trichuris* spp.), amphistomes, cestodes (*Moniezia* spp.), and oocysts of protozoans (coccidians). These observations comply with the past records [19–22].

The literature states that nematode infections and coccidial infections in cattle are considered highly parasitic if they exceed ≥500 eggs per gram (epg) and a count of ≥5000 oocysts per gram of feces, respectively [23, 24]. Fecal egg count (FEC) of 100 eggs per gram (epg) or more from* Bunostomum *species is likely to indicate severe damage, whereas a count of 500 epg of* Cooperia* species is expected to produce mild helminthosis in cattle [25]. Based on the above, the present study indicated very high parasitic infections in cattle for nematode and coccidial oocysts. The average FEC of* Trichuris *spp.,* Bunostomum *spp., and coccidian oocysts were noted as 7333 epg, 3333 epg, and 2000 epg, respectively, especially in the farms which were not having a proper treatment and investigations. However, cattle reared under the investigation of the Veterinary Investigation Centre also showed an average count of 8888 coccidial oocysts per gram of feces, but they were negative for nematode parasites.

It is interesting to note that coccidian parasites were the only abundant parasite identified from treated farms. The reason for this deviation may be due to the development of a higher resistance of Coccidia against the standard treatment protocol for cattle at 21-day, 3-month, 6-month, and 12-month intervals, respectively, with a standard mixture of Albendazole and Fenbendazole based on the body mass. Most farmers (n=100) in this study were registered under the Veterinary Investigation Centre and reared cattle in household farms. Therefore, restrictions in open grazing and proper management practices may be the reason for low prevalence of gastrointestinal parasites among treated cattle. Their control is often achieved by prophylactic use of anthelmintic treatments.

The results of this study indicated that the highest percentage of parasitic infections accounting for 46.67% (n=14) was from nontreated cattle. The statistical analysis indicated that the treatment status has significantly influenced the gastrointestinal parasitic infections (*P* =0.01 at 5% level of significance). Adding more information to this observation, prevalence of gastrointestinal parasites has been reduced along with the deworming treatment procedures over time (veterinary surgeon at the Veterinary Investigation Centre, Welisara, Pers.Comm.). According to Rajakaruna and Warnakulasooriya [3], deworming and management practices lead to low prevalence of gastrointestinal parasites.

However, most of the cattle in nontreated farms were open grazing animals and they were almost never treated for any GI infections. Grazing often encourages entering of different parasitic stages into the digestive tract of cattle through oral ingestion [3]. Fecal egg counts are highly important as an indicator to decide the period that the cattle have to be given deworming treatments. This can also be used after deworming treatments to investigate the effectiveness of a particular anthelmintic. Therefore, unnecessary costs of veterinary services and drugs can be reduced.

When using fecal egg counts, there are some limitations to determining the significance of the prevalence of flukes. The number of parasitic eggs per gram of feces is influenced by the fecal consistency, total amount of feces produced, and time of the day feces were collected. When the feces are dried, the parasitic eggs within the feces will be more concentrated.

The severity of gastrointestinal parasitic infections can be due to the vulnerability of animals to internal parasites and the poor immunity. The prevalence rate and clinical diseases may vary, based on different environmental factors in different areas. The high prevalence of gastrointestinal nematodes and coccidian oocysts has been reported in tropical regions including Sri Lanka, with prevalence rates ranging from 20 to 96% [13].

During this study the prevalence of parasitic infections was higher in buffaloes than cows. Some previous studies have indicated more than 40% prevalence of GI parasites among buffaloes compared to that of cows [23, 24, 26, 27]. However, some studies have specified more prevalence rates of GI parasites among cows than in buffaloes [26]. The variation in early findings might be due to the difference in the number of fecal samples examined, period of study, and geoclimatic conditions that favor the survival of infective stages of the parasites and of intermediate hosts, management conditions, and deworming practices. In addition, the variation in the prevalence of GI parasites in cows and buffaloes may be attributed to differences in feeding and general habitats of the two species [28].

Overall, sex-wise prevalence of GI parasites was higher in males when compared to that of females among both cows and buffaloes. The higher percentages of infection in males cannot be explained exactly, but it might be due to the neglected attitude of the farmers toward the management of male animals since many of the farms target milk production thereby focusing more on the health of females. These findings are in agreement with several other studies from different corners of the world [29–31].

According to Pfukenyi et al. [32] the susceptibility and pathogenicity of GI infections are greater in young animals than the matured ones. The present study reveals that most GI parasites were higher in calves and yearlings than in elderly cows. In case of buffaloes, the yearlings were the most susceptible followed by calves. Therefore, the findings of the current study suggest the fact that younger stages of both cows and buffaloes are more susceptible to GI parasites than the elderly stages. A recent study conducted in Ethiopia has also reported a similar finding, where the younger animals remained more susceptible than adults [33]. On the contrary, the increase in the prevalence of GI parasites with age has also been reported by several other researchers [34, 35]. However, the causes for variations in the prevalence of parasites at different age groups are difficult to explain, but they might be due to an immunological status of the animals, difference in the grazing area, and management conditions [33].

A significantly higher proportion of calves were infected with Coccidia than other age groups. This may be due to the fact that high humidity and moderate temperature facilitate the survival and sporulation of the oocysts. As their immunity is also lower than the adult cattle, calves may be more susceptible to coccidian infections [30]. In addition,* Moniezia *spp. were seen in elderly cattle. These reared cattle are pasture grazing animals. According to Irie et al.,* Oribatula sakamorii *(Oribatid mites) act as the intermediate host of* Moniezia *spp. [36]. Oribatid mites mainly live in the soil containing low biomass, such as those at farming fields, sandbreak plantations along beaches, greenbelts of urban areas, and organic matter, especially in cattle bedding litter. Therefore, larvae of the mite found in soil or litter may also be detected in the rectal feces of a cow, suggesting that the cattle at the farm had ingested them through oral ingestion. In this study, the exact presence of Oribatid mites in the farms is not known. Perhaps it might be one of the reasons for* Moniezia *spp. infections. The yearlings of buffaloes had the highest infections, followed by calves. Buffaloes were infected with* Trichuris *spp. and* Paramphistomum.* None of the other parasite stages could be observed among them. According to Bilal et al. [30], calves on grazing are heavily infected with GI parasites compared to stall fed calves and the male buffalo calves are more affected than female calves.

The highest GI infection rate (31.43%) was observed in Kelaniya, followed by Welisara (9.32%). According to the distribution pattern, amphistomes were observed from the cattle, which were at Kelaniya, while* Moniezia *spp. were found only at the Welisara area. The difference in prevalence of GI parasitic infections might be due to variation in geoclimatic conditions of these areas of study. The present study areas are located in the wet zone of Sri Lanka with a high species diversity of aquatic snails [37]. Therefore, these snails can act as the possible intermediate host for digenean trematodes. On the other hand, temperature directly affects the cercarial output owing to both the stimulating effect of temperature increasing the emergence from the snail and the acceleration of cercarial production within the snail host [38].

Overall, the present investigation indicated that the treatment status, age, gender, and spatial location were significantly influencing the gastrointestinal parasitic infections among cattle in the selected farms located in Gampaha District of Sri Lanka. This survey also highlights how the deworming and management practices of cattle affect the prevalence of parasitic infections. Therefore, periodical monitoring of the prevalence of GI parasites among farm animals is essential, in order to achieve the expected goals in deworming activities, rational use of anthelminthic drugs, and proper farm management.

## 5. Conclusion

Hookworms (*Bunostomum* spp.), whipworms (*Trichuris* spp.), amphistomes, cestodes (*Moniezia* spp.), and protozoans (coccidians) were diagnosed as gastrointestinal (GI) parasites from the cattle. The effect of treatment status was significantly correlated with the prevalence of GI parasitic infections in the study population. Males carried significantly higher GI parasites than females. Buffaloes showed significantly higher level of susceptibility to GI parasites than cows. The yearlings and calves of both cows and buffaloes had the highest rate of GI parasitic infections compared to adult animals. GI parasites in cattle population varied with spatial locations among the selected areas of Gampaha District. Future investigations are necessary to evaluate the economic impact of GI parasites in the study areas.

## Figures and Tables

**Table 1:** Percentage of GI parasitic infection rates among cattle at different treatment status.

**Treatment status**	**Percentage of infected cattle (**%**)**	***P *value** **(5**%** level of significance)**
Treated (N=100)	3 (n=3)^a^	0.01
Partially treated (N=33)	15.15 (n=5)^b^	
Nontreated (N=30)	46.67 (n=14)^c^	

**Note**: N = total number of cattle in the study population; n = number of infected cattle with GI parasites. Different superscript letters in the column indicate significant differences indicated by the General Linear Model (GLM) followed by Tukey's pairwise comparison.

**Table 2:** Parasite-wise prevalence of GI parasites in cows and buffaloes.

**Parasite and stage**	**Cows**	**Buffaloes**	**Overall**
	**(N=147)**	**(N=16)**	**(N=163)**
*Bunostomum* eggs	1.36% (n=2)	0% (n=0)	1.23% (n=2)
*Trichuris* eggs	4.76% (n=7)	12.25% (n=2)	5.52% (n=9)
*Paramphistomum *eggs	1.36% (n=2)	18.75% (n=3)	3.07% (n=5)
*Moniezia* eggs	0.68% (n=1)	-	0.61% (n=1)
Coccidians oocysts	3.40% (n=5)	-	3.07% (n=5)

**Table 3:** Percentage of GI parasite infection based on gender.

**Type**	**Gender**	**Percentage of infected cattle (**%**)**	**Percentage of overall infection (**%**)**	***P* value** **(5**%** level of significance)**
Cows (N=147)	Male (N=28)	32.14 (n=9)^a^	11.56^a^	0.04
	Female (N=119)	6.72 (n=8)^b^	(17/147)	
Buffaloes (N=16)	Male (N=5)	60.00 (n=3)^c^	31.25^b^	
	Female (N=11)	18.18 (n=2)^c^	(5/16)	

Overall (N=163)	Male (N=33)	36.36 (n=12)^a^		0.03
	Female (N=130)	7.69 (n=10)^b^		

**Note**: N = total number in the study population; n = number of infected individuals with GI parasites. Different superscript letters in a column show significant differences (*P* < 0.05) indicated by Tukey's pairwise tests after GLM.

**Table 4:** Percentage of cattle belonging to different age groups with GI parasitic infections.

**Type**	**Age group**	**Percentage of infected cattle (**%**)**	***P *value**
Cows (N=147)	Calves (N=41)	17.07 (n=7)^c^	0.042
	Yearlings (N=56)	14.29 (n=8)^c^	
	Elderly (N=50)	4.00 (n=2)^a^	

Buffaloes (N=16)	Calves (N=3)	33.33 (n=1)^b^	0.037
	Yearlings (N=4)	50.00 (n=2)^c^	
	Elderly (N=9)	22.22 (n=2)^a^	

**Note**: N = total number in the study population; n = number of infected individuals with GI parasites. Different superscript letters in a column show significant differences (*P* < 0.05) indicated by Tukey's pairwise tests after GLM.

**Table 5:** Percentage of cattle belonging to different study areas with different GI parasitic infections.

**Location**	**Percentage of infected cattle**	**Percentage of cattle infected with GI parasites (**%**)**				
		**Coccidia**	***Bunostomum***	***Trichuris***	***Paramphistomum***	***Moniezia***
Welisara	9.32^a^	36.36	9.09	36.36	0.00	9.09
(N=118)	(n=11)	(n=4)	(n=1)	(n=4)	(n=0)	(n=1)

Ganemulla	(n=0)^b^	0.00	0.00	0.00	0.00	0.00
(N=10)		(n=0)	(n=0)	(n=0)	(n=0)	(n=0)

Kelaniya	31.43^c^	9.09	9.09	36.36	45.45	0.00
(N=35)	(n=11)	(n=1)	(n=1)	(n=4)	(n=5)	(n=0)

**Note**: Different superscript letters in the column show significant differences (*P*< 0.05) indicated by Tukey's pairwise tests after GLM.

## Data Availability

The collected data will be kept confidential. Data will not be shared in any of the sources.
